# Application of Fluorescent Protein Expressing Strains to Evaluation of Anti-Tuberculosis Therapeutic Efficacy *In Vitro* and *In Vivo*

**DOI:** 10.1371/journal.pone.0149972

**Published:** 2016-03-02

**Authors:** Ying Kong, Dong Yang, Suat L. G. Cirillo, Shaoji Li, Ali Akin, Kevin P. Francis, Taylor Maloney, Jeffrey D. Cirillo

**Affiliations:** 1 Department of Microbiology, Immunology, and Biochemistry, University of Tennessee Health Science Center, Memphis, Tennessee, United States of America; 2 Department of Microbial Pathogenesis and Immunology, Texas A & M Health Science Center, Bryan, Texas, United States of America; 3 Caliper Life Sciences, PerkinElmer, Waltham, Massachusetts, United States of America; Infectious Disease Research Institute, UNITED STATES

## Abstract

The slow growth of *Mycobacterium tuberculosis* (*Mtb*), the causative agent of tuberculosis (TB), hinders development of new diagnostics, therapeutics and vaccines. Using non-invasive real-time imaging technologies to monitor the disease process in live animals would facilitate TB research in all areas. We developed fluorescent protein (FP) expressing *Mycobacterium bovis* BCG strains for *in vivo* imaging, which can be used to track bacterial location, and to quantify bacterial load in live animals. We selected an optimal FP for *in vivo* imaging, by first cloning six FPs: tdTomato, mCherry, mPlum, mKate, Katushka and mKeima, into mycobacteria under either a mycobacterial Hsp60 or L5 promoter, and compared their fluorescent signals *in vitro* and *in vivo*. Fluorescence from each FP-expressing strain was measured with a multimode reader using the optimal excitation and emission wavelengths for the FP. After normalizing bacterial numbers with optical density, the strain expressing L5-tdTomato displayed the highest fluorescence. We used the tdTomato-labeled *M*. *bovis* BCG to obtain real-time images of pulmonary infections in living mice and rapidly determined the number of bacteria present. Further comparison between L5-tdTomato and Hsp60-tdTomato revealed that L5-tdTomato carried four-fold more tdTomato gene copies than Hsp60-tdTomato, which eventually led to higher protein expression of tdTomato. Evaluating anti-TB efficacy of rifampicin and isoniazid therapy *in vitro* and *in vivo* using the L5-tdTomato strain demonstrated that this strain can be used to identify anti-TB therapeutic efficacy as quickly as 24 h post-treatment. These *M*. *bovis* BCG reporter strains represent a valuable new tool for evaluation of therapeutics, vaccines and virulence.

## Introduction

Tuberculosis (TB) is a major cause of morbidity and mortality worldwide [1]. The challenges faced in TB control are: lack of an effective vaccine, emergence of multidrug-resistant TB globally, and increasing cases of *Mycobacterium tuberculosis* and HIV co-infection in many regions of the world [2, 3]. A more effective vaccine and new antimicrobial agents are urgently needed. The causative agent of TB, *M*. *tuberculosis*, grows very slowly in culture. It takes weeks to obtain visible colonies on agar plates to determine colony forming units (CFU) after inoculation. Using non-invasive real-time optical imaging technologies to monitor the bacterial burden and disease progress in small animals would greatly accelerate research in TB. When *in vivo* optical imaging is used to estimate bacterial number, the bacteria within the site of infection can be quantified immediately based on the level of fluorescent or bioluminescent signal they produce, rather than waiting for CFU, a tedious procedure that requires weeks to obtain data.

Optical imaging systems have been successfully used in real-time monitoring of tumorigenesis and a growing number of infectious diseases [4–11]. Both bioluminescent imaging and fluorescent imaging probes have been developed for TB research [10–19], a number of which have been applied to *in vivo* imaging of *M*. *tuberculosis* [11, 12, 14, 18, 19]. Fluorescent proteins (FPs) have been used widely in cell and animal imaging to track cellular movement in embryogenesis and inflammatory processes [20, 21], to monitor pathogen migration in host [22–24], and to study carcinogenesis processes in live animals [4, 25–28]. Imaging fluorescent signals in deep tissues of animals requires that the FP has excitation and emission wavelengths within or at least close to the near-infrared (NIR) window, which is 600–900 nm, because hemoglobin and water have their lowest absorption coefficients in the NIR window [29]. Several FPs have excitation and emission wavelengths close to this NIR window, with red or far-red colors ranging from 581 nm to 655 nm [30].

After carefully comparing excitation and emission wavelengths, brightness, and photostability, we selected mPlum [31], mKate, Katushka [28], Keima [32], mCherry, and tdTomato [33] to clone into mycobacterial strains, and evaluate their sensitivity for detecting mycobacteria *in vitro* and *in vivo*, using a multi-mode spectrometer and whole animal imaging system, IVIS (PerkinElmer, Waltham, MA). The FP expressing strain having the highest sensitivity of detection was successfully applied to evaluation of the efficacy of anti-TB therapy in bacterial culture and in live animals.

## Materials and Methods

### Plasmid Construction

The plasmids carrying tdTomato, mCherry and mPlum genes (pRSETB-tdTomato, pRSETB-mCherry, and pBAD-mPlum) were acquired from Dr. Rodger Y. Tsien’s laboratory. pmKeima-Red-S1 was from Amalgaam Co.; pTagFP635-C mKate and pTurboFP635-C Katushka were purchased from Evrogen Co. To construct plasmids expressing tdTomato or mCherry under Hsp60 promoter, pRSETB-tdTomato or pRSETB-mCherry DNA was cut with *Nhe*I and *Sca*I. After gel purification to separate the DNA sequences containing tdTomato or mCherry, the DNA fragments were ligated into the *Nhe*I / *Sca*I cut linear fragment of the *Escherichia coli-*mycobacterium shuttle plasmid pJDC89 [34] that carries the Hsp60 promoter to generate pJDC122 (Hsp60-tdTomato) or pJDC124 (Hsp60-mCherry). To construct plasmids expressing mPlum, Keima, mKate or mKatushika under Hsp60 promoter, these four FP genes were amplified with PCR using an up-stream primer containing *Nhe*I site at 5’ end and a down-stream primer containing *Pac*I site at 5’ end. The PCR products were then cut with *Nhe*I / *Pac*I, and ligated with the *Nhe*I / *Pac*I digested linear fragment of the pJDC89 to generate pJDC128 (Hsp60-mPlum), pJDC140 (Hsp60-Keima), pJDC130 (Hsp60-mKate), and pJDC131 (Hsp60-Katushka). The plasmids constructed and primers used for PCR are listed in Table 1.

**Table 1:** Plasmids and primers.

FP	Plasmids	Primers
tdTomato	pJDC122	F: 5’- TATAAAGCTTGGATCCATGGTGAGCAAGG-3’
	pJDC60	R: 5’-TATAGGTACCGAATTCTTACTTGTACAGCTCGTCC-3’
mCherry	pJDC124	F: 5’- TATAAAGCTTGGATCCATGGTGAGCAAGG-3’
	pJDC171	R: 5’-TATAGGTACCGAATTCTTACTTGTACAGCTCGTCC-3’
mPlum	pJDC128	F: 5’- TATAGCTAGCGCCACCATGGTGAGCAA-3’
		R: 5’-TATATTAATTAATAGGCGCCGGTGGAGT-3’
mKate	pJDC130	F: 5’-TATAGCTAGCATGGTGTCTAAGGGCGAAGA-3’
		R: 5’-TATATTAATTAAATTAAGTTTGTGCCCCAGTTTG-3’
Katushka	pJDC121	F: 5’-TATAGCTAGCCCATGGTGGGTGAGGATAG-3’
		R: 5’-TATATTAATTAAGCTGTGCCCCAGTTTGCTAG-3’
Keima	pJDC140	F: 5’-TATAGCTAGCCATGGTGAGTGTGATCGCTAA-3’
		R: 5’-TATATTAATTAAGTTAACCGAGCAAAGAGTGGC-3’

To construct the plasmids expressing tdTomato or mCherry under the mycobacterial phage L5 promoter, we first PCR amplified the two genes from pRSETB-tdTomato or pRSETB-mCherry using an up-stream primer containing *Hind*III site at 5’ end and a down-stream primer containing *Kpn*I site at 5’ end (Table 1). The primer pair was the same for both genes, because tdTomato is a tandem dimer of mCherry. We then cut pFJS8, an *E*. *coli-*mycobacterium shuttle plasmid containing the L5 promoter [35], with *Hind*III / *Kpn*I, and separated the larger fragment by gel purification. The *Hind*III / *Kpn*I digested PCR products were also gel purified to get tdTomato and mCherry specific fragments, and then ligated into this *Hind*III / *Kpn*I cut pFJS8 linear fragment, to form pJDC60 (L5-tdTomato) or pJDC171 (L5-mCherry).

### Strains and Growth Conditions

Plasmids were transformed into *M*. *smegmatis* and *M*. *bovis* BCG. Bacteria were grown in 7H9 broth (Difco, Detroit, MI) supplemented with 0.5% glycerol, 10% OAD (oleic acid dextrose complex without catalase) and 0.05% Tween 80 (M-OADTW broth), or Middlebrook 7H9 supplemented with 10% OAD and 15 g/l Bacto agar (M-OAD agar, Difco) or on 7H11 selective agar (Difco). When necessary, media and plates were supplemented with 80 μg/ml hygromycin or 25 μg/ml kanamycin. Frozen stocks were prepared from strains for experiments by growth standing at 37°C until an OD_600_ = 0.5 and stored in aliquots at -80°C until use.

### Real-time qPCR

Reverse transcription was carried out with the SuperScript First-Strand Synthesis System for RT-PCR based on Invitrogen’s protocol. Briefly, RNA/primer mixture in each tube was prepared as: total RNA 150 ng, random hexamers (150 ng/ul) 1 ul, 1.25 mM dNTP mix 8 μl, and adding DEPC H_2_O to 12 μl. The samples were incubated at 65°C for 5 min and then on ice for at least 1 min. Reaction master mixture was prepared as: 5x RT buffer 4 μl, 0.1 M DTT 2 μl, and RNAaseOUT 1 μl. The reaction mixture was added to the RNA/primer mixture, mix briefly, and then place at 37°C for 2 min. One μl (10,000 units) of Super Script III RT was added to each tube, mixed and incubated at 25°C for 10 min. The tubes were then incubated at 37°C for 50 min, heat inactivated at 70°C for 15 min, and then chilled on ice. One μl RNase H was added into the tubes and tubes were incubated at 37°C for 20 min. The first strand cDNA was stored at -20°C until use for real-time PCR. For real-time qPCR, each primer (forward or reverse) concentration in the mixture was 12.5 pmol/μl. 0.4 μl of each primer, Supermix 10 μl, Rox 25 uM 0.4 μl, cDNA or DNA template 2 μl, and H_2_O were mixed in a total 20 μl volume. Real time qPCR was set up following PCR program on StepOnePlus™ Real-Time PCR System as: 1) 50°C 2 min, 1 cycle; 2) 95°C 2 min, 1 cycle; 3) 95°C 15 s and then 60°C 1 min, 40 cycles to measure the fluorescence. Following the last cycle, the instrument generated a melting curve by ramping from 60°C to 95°C at 0.2°C/s and continuously measuring the fluorescence. 4) 95°C 15 s; and 5) 60°C 1 min, 95°C 15 s, 1 cycle. 16s rRNA was used as an internal control. Primer sequences for tdTomato: F: 5’-CAAGCTGAAGGTGACCAAGG-3’; R: 5’-GTGATGAACTTCGAGGACGG-3’. Primer sequences for 16s rRNA: F: 5’-CCGCAAGRCTAAAACTCAAA-3’; R: 5’-TGCACACAGGCCACAAGGGA-3’. Reactions with amplification cycle threshold values of between 20 and 35 were considered successful and were analyzed with DNA melting curves.

### Macrophage Infection Assays

The murine macrophage cell line J774A.1 (ATCC TIB67) was maintained at 37°C and 5% CO_2_ in high glucose Dulbecco’s Modified Eagle Medium (DMEM; Gibco, Bethesda, MD) supplemented with 10% heat-inactivated fetal bovine serum (FBS, Gibco) and 2 mM L-glutamine. J774A.1 cells were infected in a similar manner to that described previously [36]. J774A.1 cells were seeded at 2.5x10^5^ cells/well in 24-well tissue culture plates or 1x10^5^ cells/well in 8-well chamber slides and incubated overnight at 37°C in DMEM. The media was then replaced just prior to infection with 0.2 ml of media that contains 2.5 x 10^6^ bacteria, equivalent to a multiplicity of infection (MOI) of approximately 10 (bacteria/cell). The bacteria were incubated with the cells for 30 min at 37°C and washed twice with warm PBS, then the infected cells were incubated with DMEM plus 10% FBS and 200 μg/ml amikacin for 2 h at 37°C. The cells in a triplicate set of wells were then prepared for microscopy or entry assays.

### Mouse Infections

All animal experiments in this study were approved by Texas A&M University Institutional Animal Care and Use Committee. Five to seven week old female BALB/c mice were obtained from Jackson Laboratories. All animals were housed in polycarbonate microisolator cages in a controlled environment with 12h light / 12h dark cycle, ~18–23°C, and 40–60% humidity. There were four animals in each experimental group. Animals were assigned randomly to experimental groups, allowed to acclimate to the facilities for one week and fed commercial chow with low chlorophyll content and tap water *ad libitum*. Portions of lungs were homogenized in PBS, and dilutions were plated on 7H11 selective media to determine numbers of bacteria present at each time point. Sub-cutaneous infections were carried out by suspending the appropriate concentration of bacteria in 50 μl of saline prior to inoculation directly beneath the skin. Bacterial numbers were confirmed at 24 h post-inoculation by harvesting the region of the skin and homogenization in PBS followed by dilution and plating on 7H11 selective media to determine the colony forming units present. Alternatively, mice were infected intratracheally with *M*. *bovis* BCG, as described previously [37, 38]. Briefly, mice were anesthetized with ketamine and xylazine mixture by intraperitoneal injection, and were put in a ventral position on a stand. The mouse tongue was pull out of the mouth with a forceps. After seeing the larynx opening with an otoscope, a catheter with the guide wire running through was carefully placed into the larynx. The guide wire was then removed, and 50 μl of bacteria in saline was pipetted into the hub of the catheter. The bacteria were then flushed into lungs with 50 μl of air from a 1 ml syringe. Four mice per group were imaged and sacrificed for necropsy to determine thresholds of detection. Antibiotic treatment was carried out with four mice per group, randomly allocated, by administration of RIF plus INH intraperitoneally daily at 10 mg kg^−1^ animal body weight. At each time point a group of animals in each treatment category were imaged, necropsied, lungs imaged and homogenized for CFU determination by plating dilutions of tissue homogenates. Each experiment was independently performed twice.

### Imaging Tuberculosis Infections

Mice were anesthetized with isofluorane and imaged in an IVIS Spectrum (Caliper Life Sciences) with and without filters for fluorescence. Photographic images were directly overlaid with matching fluorescent images for all mice. Structural images were taken in all cases where fluorescence molecular tomography was used. Wavelength-resolved spectral imaging was carried out to image fluorescent protein-expressing BCG in mice. In the case of tdTomato, the excitation wavelength was 535 nm and emission was collected in 20 nm increments from 540 nm to 660 nm; for mCherry the excitation wavelength was 570 nm and emission was collected in 20 nm increments from 580 nm to 680 nm. Each acquisition was taken for 2–4 s with f-stop one and medium binning. Images were analyzed with Living Image Software v4.1 using spectral unmixing algorithms to remove autofluorescence, as described by the provider, with one of the resulting channels locked to fit the emission spectrum of the appropriate fluorophore (tdTomato or mCherry).

### Confocal Fluorescent Microscopy

#### J774 A.1 cell infection

Cells were seeded in 8-well chamber slides with 1 x 10^5^ cells per well in 200 μl of DMEM plus 10%FBS overnight at 37°C in 5% CO_2_. The medium was removed and the tdTomato expressing *M*. *bovis* BCG was added to each well at an MOI of 10 (bacteria per cell) in 200 μl of medium. The bacteria were co-incubated with the cells for 30 min, washed twice with PBS to remove extracellular bacteria and medium plus 200 μg/ml amikacin added for 2 h at 37°C to select for intracellular bacteria. The cells were then stained with 10 μg/ml DAPI in PBS by incubation for 5 minutes at room temperature, washed twice with PBS and fixed in 4% paraformaldehyde in PBS for 30 min at room temperature. After washing with PBS the slides were dried and mounted for viewing by confocal fluorescent microscopy.

#### Cryosections of lungs from infected mice for microscopy

Lungs were removed from intratracheally infected mice, washed with PBS twice, and fixed with 4% paraformaldehyde for 24 h. The lungs were then washed with PBS twice and transferred into 30% sucrose until the lungs sank. Lung tissue was placed in the cryomold, overlaid with O.C.T. compound (Sakura Finetek USA Inc.), oriented and frozen quickly on dry ice. Cryosectioning was conducted with the frozen lung tissue, and the lung tissues on the slides were then stained with DAPI. After washing with PBS the slides were dried and mounted for viewing by confocal fluorescent microscopy.

### Statistical Analyses

The significance of the results was determined using the Student’s t-test or ANOVA, as appropriate. *P* values of < 0.05 were considered significant. In figures, * represents *P*<0.05; ** represents *P*<0.01; and *** represents *P*<0.001.

## Results

### 1. tdTomato is the brightest fluorescent protein expressed by mycobacteria in culture

**Fig 1:**
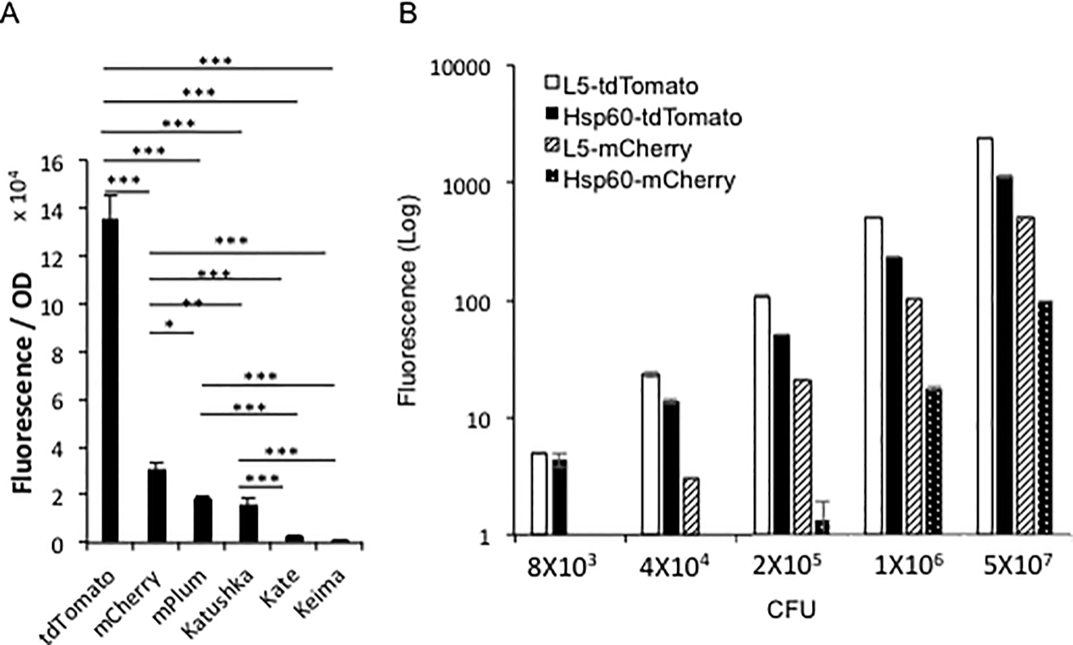
*In vitro* comparison of the brightness among different fluorescent protein-expressing mycobacterial strains. A. Comparison of the brightness among different fluorescent protein expressing mycobacterial strains in culture medium. Different fluorescent protein expressing *M*. *bovis* BCG strains were cultured in MOAD medium to OD_600_ 0.5~1, and then the fluorescent signal of them were detected with a multimode reader at the optimal wavelength condition for each FP. For each FP expressing strain, fluorescence signal was calculated by subtracting the background fluorescence of the strain carrying the backbone vector for the FP. The Y-axis value was calculated mean of fluorescence of triplicates divided by their OD at 600 nm. The error bars are standard deviations. One-way ANOVA test was conducted for assessing overall differences among groups, and Turkey’s multiple comparison tests were applied to assess differences between two groups. * *P*<0.05; ** *P*<0.01; and *** *P*<0.001. B. Comparison of the brightness among *M*. *bovis* BCG-L5-tdTomato, *M*. *bovis* BCG-Hsp60-tdTomato, *M*. *bovis* BCG-L5-mCherry, and *M*. *bovis* BCG-Hsp60-mCherry. Five concentrations for each strain of bacteria were loaded into a 96-well plate. The fluorescence of samples were compared by a multimode reader with 530 nm excitation and 590 nm emission wavelengths for tdTomato; 570 nm excitation and 620 nm emission wavelengths for mCherry. Y-axis is in log scale. Data represent one of at least three independent replicate experiments. The error bars are standard deviations.

Six FPs, tdTomato, mCherry, mPlum, mKate, Katushka and Keima, were cloned into *M*. *smegmatis*, and *M*. *bovis* BCG downstream from a mycobacterial Hsp60 promoter. The fluorescent signal was measured under the optimal excitation and emission wavelengths for each FP (tdTomato: ex: 530 nm, em: 590 nm; mCherry: ex: 570 nm, em: 620 nm; mPlum, mKate, Katushka: ex: 570 nm, em: 650 nm; and Keima: ex: 485 nm, em: 620 nm). Absorbance at 600 nm was measured for each strain to estimate bacterial numbers present. The brightness of each strain was calculated by dividing relative fluorescence units of the strain by its optical density (OD) at 600 nm (Fig 1A). In both BCG and *M*. *smegmatis*, tdTomato was the brightest FP among the selected FPs, followed by mCherry, mPlum, Katushka, mKate, and Keima.

We evaluated the impact of signal interference due to mammalian tissue by comparing tdTomato and mCherry in Eppendorf tubes filled with *M*. *smegmatis* expressing Hsp60-tdTomato or Hsp60-mCherry, and covering the tubes with different thicknesses of sliced ham. These eppendorf tubes covered with sliced ham were then imaged to evaluate the relative fluorescent signal displayed by each strain (S1 Fig). *M*. *smegmatis* expressing tdTomato was 5.6-fold brighter than the one expressing mCherry when using the same number of CFU.

The tdTomato and mCherry were then cloned under expression from a L5 promoter and compared with strains expressing tdTomato or mCherry from the Hsp60 promoter. The strains expressing FPs from the L5 promoter are brighter than the ones using the Hsp60 promoter, when fluorescence was measured with a multimode reader (Fig 1B).

### 2. Mycobacterial CFU can be estimated by fluorescence *in vitro*

We evaluated the ability to quantify bacterial CFUs using the fluorescent signals from the FP expressing strains. This was accomplished by making dilutions of the bacteria and loading 100 μl of each concentration into a 96-well plate. The fluorescence from the four FP expressing strains correlated very well with bacterial CFUs, R^2^ = 1 for L5-tdTomato and L5-mCherry; and R^2^ = 0.999 for Hsp60-tdTomato and Hsp60-mCherry, indicating that we can accurately estimate bacterial CFU with fluorescence.

The multimode reader could detect ≥8,000 CFU of BCG-L5-tdTomato and BCG-Hsp60-tdTomato (Fig 1B), but it could not detect the two mCherry strains at the same CFU. BCG-L5-mCherry could be detected at ≥40,000 CFU, while BCG-Hsp60-mCherry could be detected at ≥200,000 CFU.

**Fig 2:**
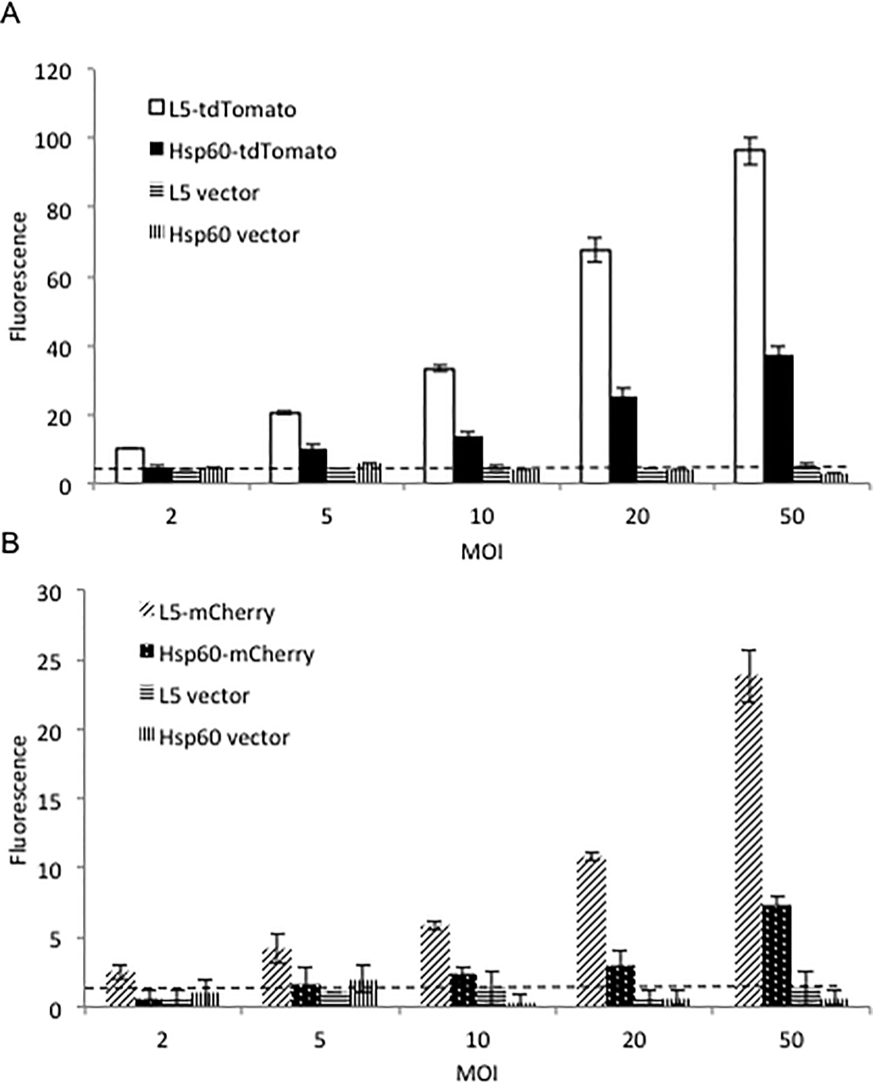
Comparison of strains expressing fluorescent proteins under L5 and Hsp60 promoter expression in infected cells. J774A.1 cells were infected with various multiplicities of infection (MOI) of the BCG strains expressing L5-tdTomato or Hsp60-tdTomato (A); L5-mCherry or Hsp60-mCherry (B). Fluorescence was measured with a multimode reader with excitation wavelength 530 nm and emission wavelength 590 nm for tdTomato and excitation wavelength 570 nm and emission wavelength 620 nm for mCherry. Value in Y-axis represents mean of fluorescence of triplicates divided by their OD at 600 nm. The error bars are standard deviations. Dotted lines represent background fluorescence from strains carrying backbone vectors. Data represent one of at least three independent replicate experiments.

Mycobacteria expressing tdTomato and mCherry from the two promoters were compared during infection of mammalian macrophages. J774 A.1 macrophage cell line was infected with the four FP expressing *M*. *bovis* BCG strains at five different multiplicities of infection (MOI) (Fig 2). The fluorescent signals from the intracellular BCG strains were measured with a multimode plate reader. Again, tdTomato expressing strains showed higher sensitivity, and strains with the L5 promoter for FPs displayed higher fluorescence than did the strains expressing the reporter from the Hsp60 promoter. The fluorescence levels from the four strains increased as the MOIs went higher (L5-tdTomato, R^2^ = 0.90; Hsp60-tdTomato, R^2^ = 0.94; L5-mCherry, R^2^ = 0.92; Hsp60-mCherry, R^2^ = 0.93), which should represent a quantitative measure of intracellular CFUs. These observations demonstrate that fluorescence from these strains could be used to accurately estimate intracellular bacterial numbers.

### 3. Comparison of fluorescence from mycobacteria expressing FPs during subcutaneous infection in mice

**Fig 3:**
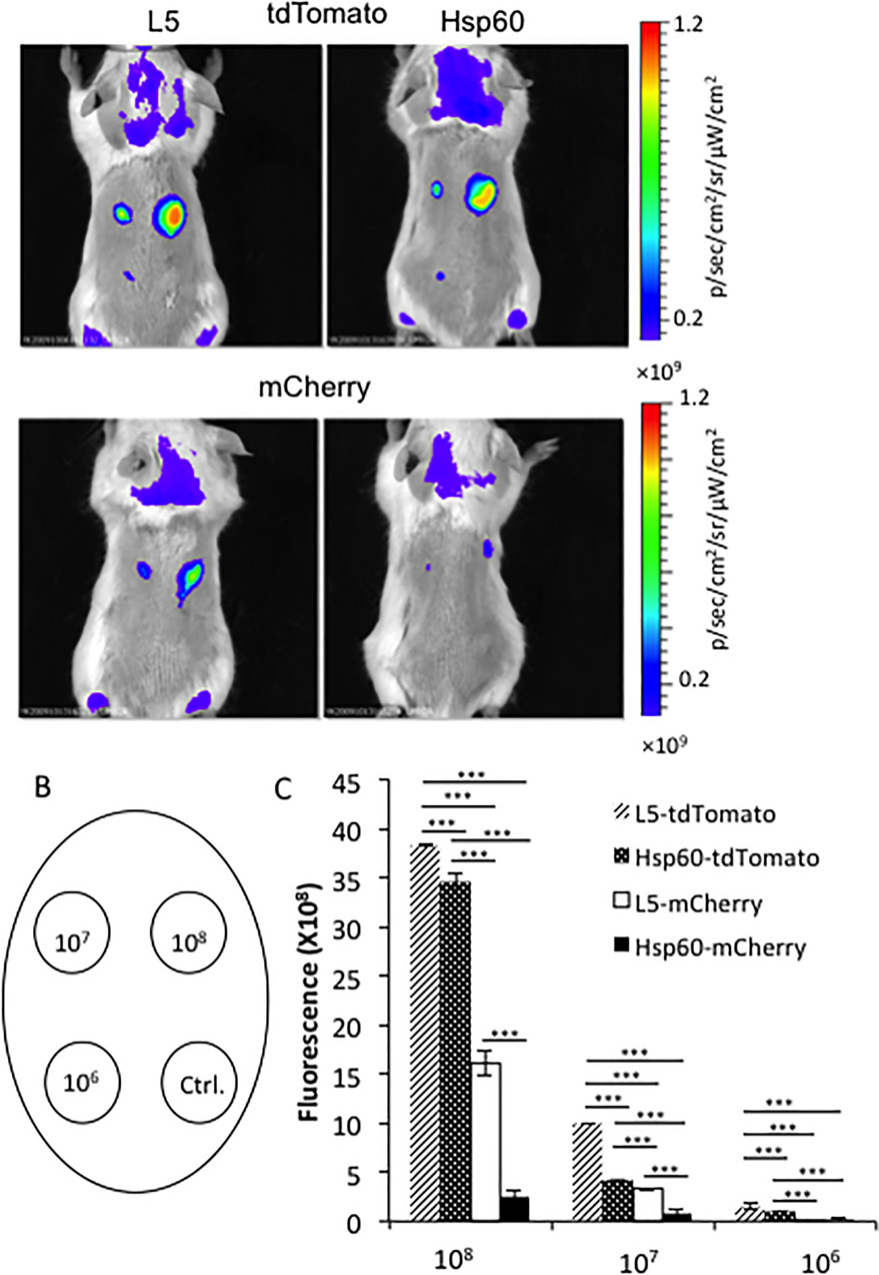
Comparison of brightness among BCG strains expressing tdTomato, or mCherry in subcutaneously infected mice. A. Images of subcutaneous infected mice with *M*. *bovis* BCG strains expressing L5-tdTomato, Hsp60-tdTomato, L5-mCherry, or Hsp60-mCherry. 10^8^, 10^7^, and 10^6^ CFU of bacteria were injected subcutaneously into BALB/c mice. Images were acquired by epi-illumination at day 1 post infection. B. The locations of the injections were shown on the map. C. Quantitative analysis of the images. Four mice were infected with technical replicates of the same culture. Values in the Y-axis represent mean of fluorescence for each dose of bacteria. The error bars are standard deviations. Data represent one of two independent replicate experiments. One-way ANOVA test was conducted for assessing overall differences among groups, and Turkey’s multiple comparison tests were applied to assess differences between two groups. * *P*<0.05; ** *P*<0.01; and *** *P*<0.001.

To select the best FP expressing strain for imaging in mammalian tissues, we conducted subcutaneous infections in mice. This model allowed us to compare fluorescence detected from different doses of bacteria, and/or different strains on the same mouse, thus background auto fluorescence is well normalized. *M*. *bovis* BCG L5-tdTomato, Hsp60-tdTomato, L5-mCherry, and Hsp60-mCherry were subcutaneously inoculated into BALB/c mice. The fluorescent signals were evaluated using epi-illumination imaging and optimal excitation and emission wavelengths for tdTomato and mCherry, respectively. Images were analyzed by spectral unmixing to separate FP specific signal from autofluorescence in the animals (Fig 3). Detailed imaging protocols are described elsewhere [37]. The BCG strain carrying the plasmid backbone without a FP was used as a negative control in each mouse. At the same CFU, the BCG-L5-tdTomato strain displays higher fluorescence than the BCG-Hsp60-tdTomato strain; and tdTomato expressing strains display significantly higher fluorescence than mCherry expressing strains. Imaging allows detection of ≥10^6^ CFU of both BCG-L5-tdTomato and BCG-Hsp60-tdTomato strains in mice after subcutaneous inoculation by epi-illumination imaging. We chose the L5-tdTomato strain for future experiments, because it showed the highest fluorescence at optimal excitation and emission wavelengths after adjusting for bacterial numbers, *in vitro* and *in vivo*.

### 4. Expression of tdTomato did not affect mycobacterium growth in culture medium, and the plasmids were stably carried by mycobacterial strains

**Fig 4:**
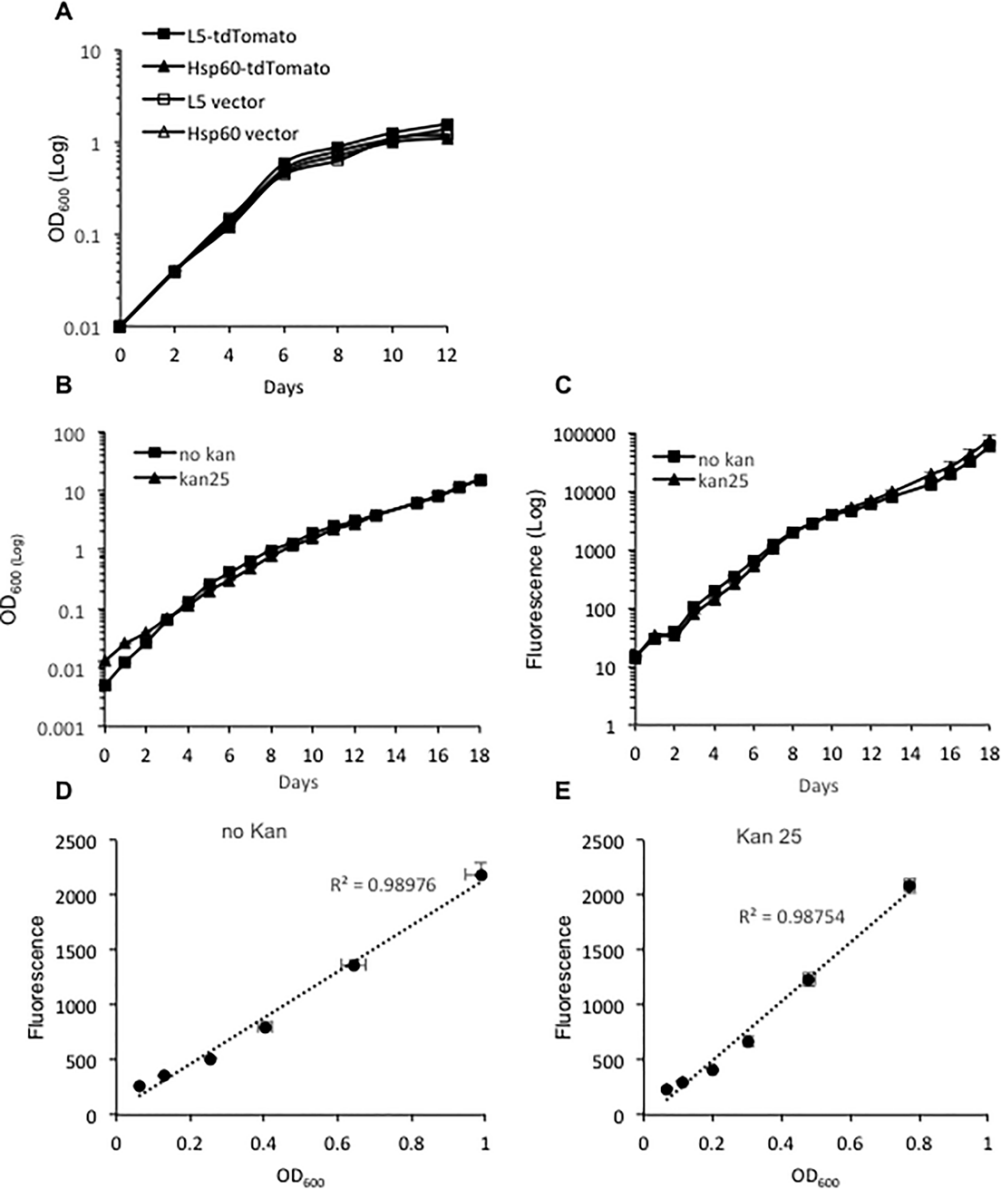
Evaluation of plasmid stability and the absence of effects on bacterial growth. A. Growth curves for *M*. *bovis* BCG strains carrying L5-tdTomato, Hsp60-tdTomato or the vector plasmids alone (no fluorescent protein expressed). B. Optical density (OD) of cultures at 600 nm in 7H9 media with (kan 25) or without (no kan) 25 μg/ml kanamycin over 18 days of culture. C. Fluorescence changes of the strain in media with or without kanamycin over 18 days of culture. D. Correlation between fluorescence and OD at 600 nm for the strain grown in medium without kanamycin. E. Correlation between fluorescence and OD at 600 nm for the strain grown in medium with kanamycin. For correlation analyses, samples for OD_600_ within the range of 0.05–1 were selected. Data represent one of at least three independent replicate experiments. Values in the Y-axis represent mean of fluorescence or OD values. The error bars are standard deviations.

Expression of a foreign gene could be toxic to the host bacteria, and thus slows down bacterial growth [19]. We evaluated whether expression of tdTomato affects mycobacterium growth. *M*. *bovis BCG* strains with plasmids expressing tdTomato under L5 or Hsp60 promoter were compared with strains carrying the vector plasmids alone. OD values at 600 nm for each strain was measured at each time point. There were no significant differences in growth curves among L5-tdTomato or Hsp60-tdTomato strains and the strains carrying vectors alone (Fig 4A). These observations suggest that expression of tdTomato does not affect bacterial growth.

Recombinant bacterial strains carrying plasmids require selective antibiotic in culture media to ensure they are maintained. We evaluated stability of the tdTomato expressing plasmid in mycobacteria without the selective pressure of antibiotic. *M*. *bovis* BCG carrying L5-tdTomato was cultured in MOAD with or without kanamycin, initially inoculated at OD_600_ 0.005 and grown for 18 days. Fluorescence and OD_600_ during culture were measured. As shown in Fig 4B–4E. The L5-tdTomato expressing strain cultured in medium without antibiotic had the similar levels of fluorescence and OD as cultured in medium with the selective antibiotic for the entire 18-day of culture, which suggests the strain can carry the plasmid stably for at least 18 days without antibiotics. Correlations between fluorescence and OD_600_ were analyzed within the range of OD_600_ from 0.05 to 1. With or without the selective antibiotic in media, the fluorescence from this strain correlates well with bacterial numbers (OD_600_), suggesting the measured fluorescence of this strain can be a good surrogate for bacterial numbers.

### 5. *In vivo* imaging mice intratracheally infected with mycobacteria expressing tdTomato

**Fig 5:**
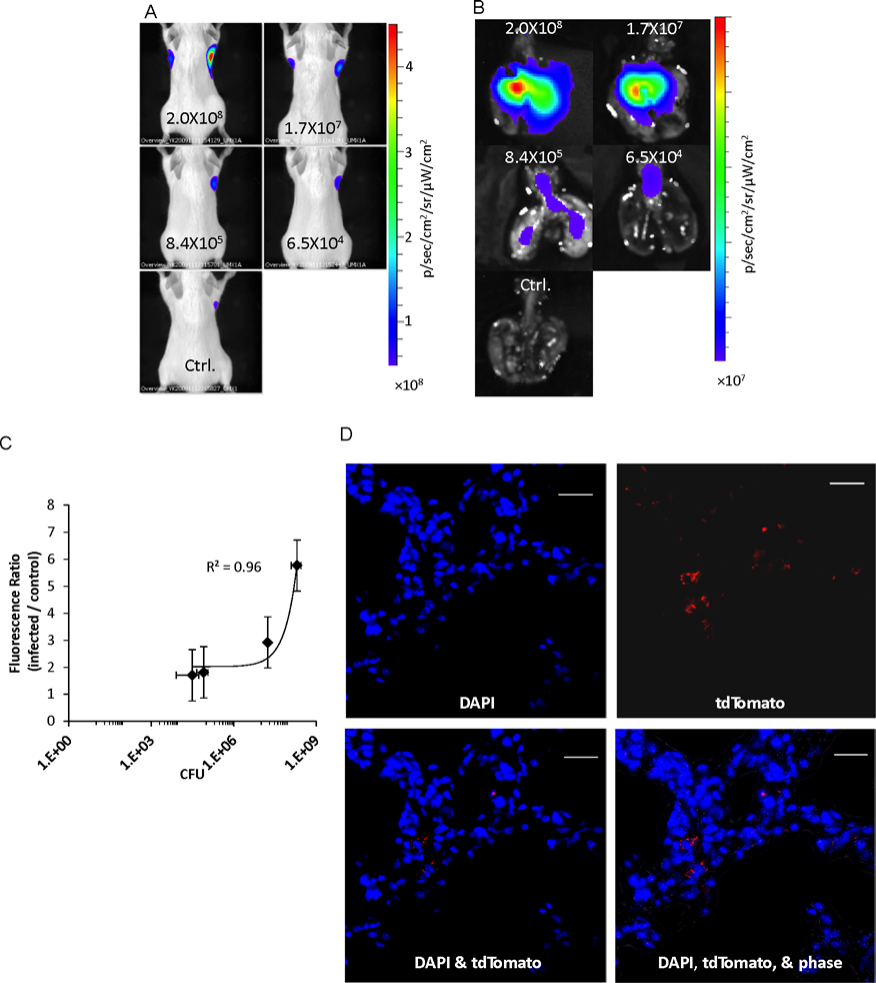
Imaging BALB/c mice infected intratrachealy with *M*. *bovis* BCG expressing L5-tdTomato. A. IVIS images of mice infected intratrachealy with the L5-tdTomato expressing strain. Images were acquired by trans-illumination at day 1 post infection. B. Images of the lungs harvested from the infected mice at day 1 post infection. Images were acquired by epi-illumination. Colony forming units (CFU) from plating dilutions on agar plates are indicated in the images. C. Quantitative analysis of images of the infected mice and the correlation between fluorescence ratios and CFUs. Four mice were infected for each dose of bacteria from technical replicates of the same culture. Data represents one of two independent replicate experiments. Value in Y-axis represents mean of fluorescence for each dose of bacteria. The error bars are standard deviations. D. Fluorescent microscopy of lung cryosections from BCG-L5-tdTomato intratracheally infected mice. Upper left: DAPI; upper right: tdTomato; lower left: merge DAPI and tdTomato; and lower right: merge DAPI, tdTomato and phase). White bars represent 20 micrometers.

We evaluated detection of the BCG-L5-tdTomato strain during pulmonary infections in mice. BALB/c mice were intratracheally infected and imaged by transillumination at day 1 post infection. The intratracheal infection model allowed us to directly deliver precise doses of bacteria, even higher doses than using aerosols are easily possible, into mouse lungs and to evaluate correlation between *in vivo* detected fluorescence and bacterial CFU. Bacterial numbers in the lungs were confirmed by conventional CFU determination. The results are shown as Fig 5A. Imaging can detect as low as 6.5 x 10^4^ CFU of BCG-L5-tdTomato during pulmonary infection using transillumination. The *in vivo* imaging results were verified with *ex vivo* imaging by epi-illumination of lungs harvested from the same mice (Fig 5B). The fluorescence correlated well with CFUs recovered after plating lung homogenates (R^2^ = 0.96, Fig 5C). The bacteria in lungs were further confirmed by fluorescence confocal microscopy on cryosections from lung tissues (Fig 5D).

### 6. Application of tdTomato expressing mycobacteria to evaluation of anti-TB therapeutic efficacy

**Fig 6:**
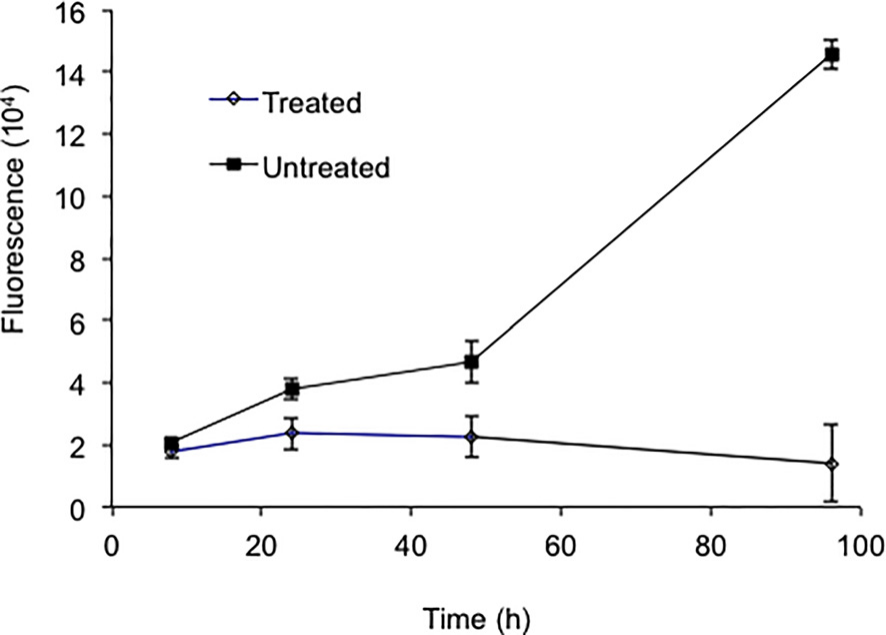
*In vitro* evaluation of anti-TB therapeutic efficacy using the *M*. *bovis* BCG L5-tdTomato strain. Fluorescence of treated or untreated *M*. *bovis* BCG L5-tdTomato strain was measured with a multimode reader. The initial CFU for the treated or untreated was 10^6^. The treated group was given 64 ng/μl rifampicin and isoniazid. At each time point, bacteria were loaded into a 96-well plates as quadruplicate samples for each group. The Y-axes represent mean fluorescence for the quadruplicates. Data represents one of two independent replicate experiments. Error bars are standard deviations. Repeated Measures Two-way ANOVA was performed to examine overall difference between the two groups (treated vs. untreated) through multiple time points. Comparison of treated with untreated group was matched at each time point. *P*<0.01.

We are interested in whether FP expressing bacteria can speed TB research involved in evaluation of candidate anti-TB therapeutic compounds. The BCG-L5-tdTomato strain was loaded into 96-well plates and treated with rifampin (RIF) and isoniazid (INH). Fluorescence changes over time were measured with a multimode reader (Fig 6) and fluorescent imager (S2 Fig) with and without treatment over 96 hours. We found that in the untreated group fluorescence increased as the bacteria replicate; whereas, the treated group displayed reduced fluorescence over time. The difference in fluorescence between treated and untreated groups was significant by 24 h treatment.

**Fig 7:**
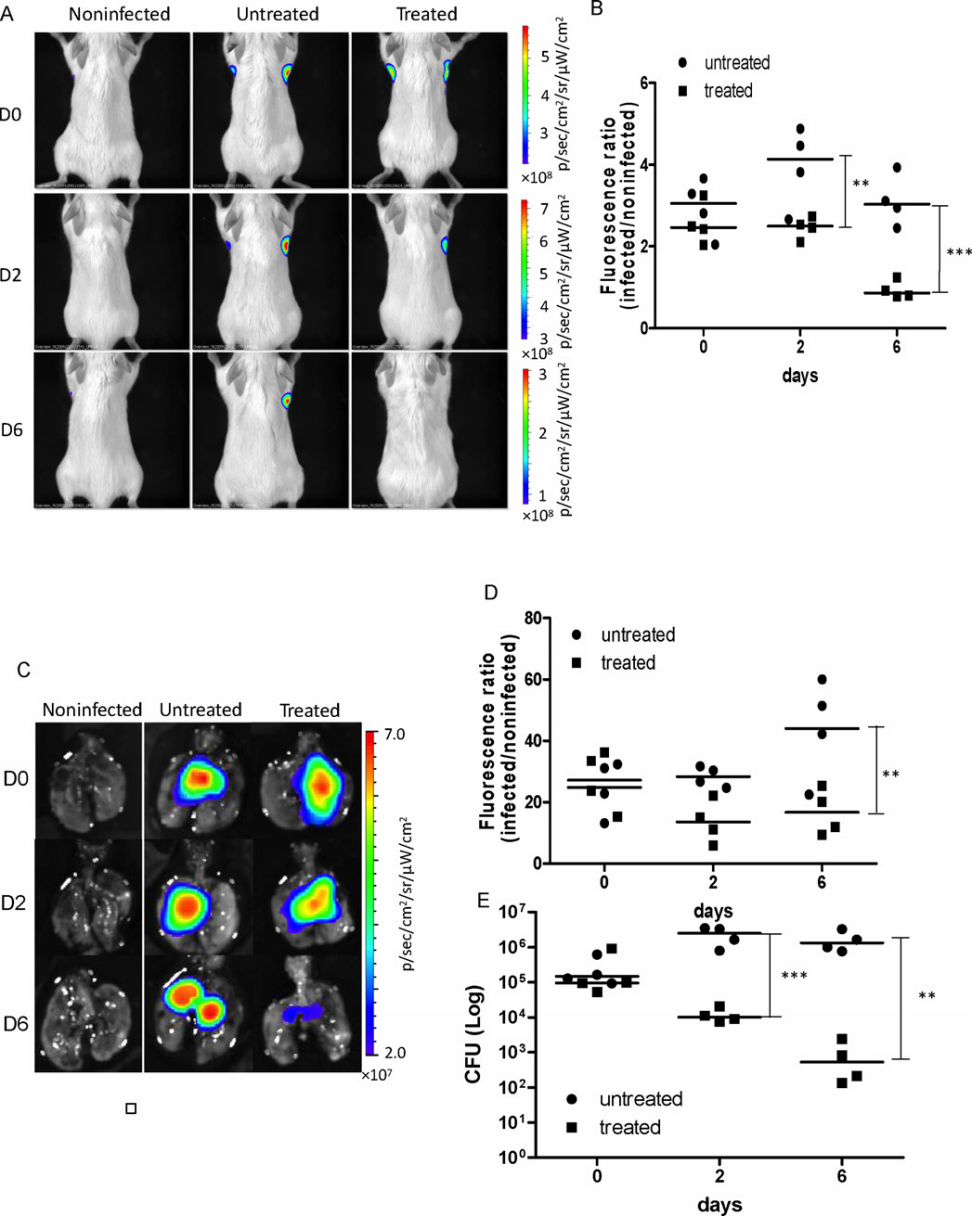
*In vivo* evaluation of anti-TB therapeutic efficacy using the *M*. *bovis* BCG L5-tdTomato strain. A. Images of infected mice treated or untreated with anti-TB therapy. Mice were infected intratracheally with *M*. *bovis* BCG with L5-tdTomato and treated or untreated with anti-TB therapy (10 μg/g rifampicin and isoniazid; intraperitoneal injection.). Images were acquired by trans-illumination at day 0, 2, and 6 post infection. B. Quantitative analysis of *in vivo* images. C. Images of lungs harvested from the mice sacrificed at day 0, 2, and 6 post infection after *in vivo* imaging. Images were acquired by epi-illumination. D. Quantitative analysis of images of harvested lungs. E. Colony forming units (CFU) in mouse lungs recovered from agar plating. Y-axis is in log scale. There were four infected mice and one uninfected mouse per group per time point. Data represents two independent replicate experiments. Two-way ANOVA was performed to examine overall difference between the two groups (treated vs. untreated) through multiple time points. Bonferroni post-hoc tests have been performed between treated and untreated groups at each time point. * *P*<0.05; ** *P*<0.01; and ***P<0.01

We also evaluated if tdTomato expressing mycobacterial strains can be applied to evaluation of anti-TB therapy *in vivo* during pulmonary infections. BALB/c mice were intratracheally infected with 10^7^ CFU of *M*. *bovis* BCG L5-tdTomato, treated with RIF and INH daily by i.p., and imaged at day 0, 2 and 6 post-treatment. Starting from day 2, the treated group displayed reduced fluorescence in the lungs (Fig 7). These observations demonstrate that a tdTomato expressing mycobacterial strain can be used to rapidly evaluate anti-TB therapeutic efficacy. Imaging is more time efficient than the previously standard approach of using conventional CFU plating methods to determine therapeutic efficacy.

### 7. The L5-tdTomato expressing *M*. *bovis* BCG strain carries more tdTomato gene copies than the Hsp60-tdTomato strain

**Fig 8:**
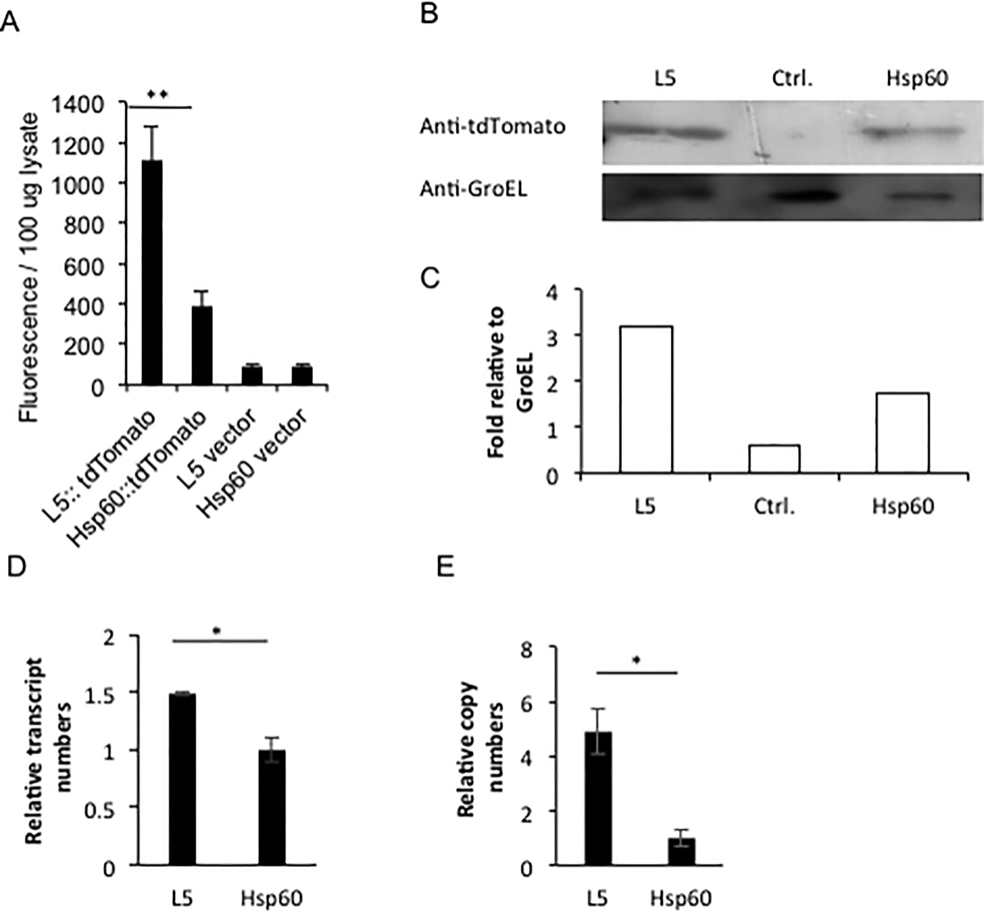
Comparison between L5-tdTomato and Hsp60-tdTomato *M*. *bovis* BCG strains in protein levels, transcript numbers and gene copies of tdTomato. A. Comparison of the fluorescence from supernatant of bacterial lysates between *M*. *bovis* BCG L5-tdTomato and Hsp60-tdTomato strains. *M*. *bovis* BCG L5-tdTomato and Hsp60-tdTomato strains were grown until OD_600_ = 0.75. 10^6^ bacteria/ml were ultrasonically lysed in 1x PBS with 0.1% Triton X-100. After centrifugation, the supernatant from the samples was compared using a multimode reader with 530 nm excitation and 590 nm emission wavelengths. B. Western Blotting to compare tdTomato protein levels expressed from an L5 promoter with that expressed from the Hsp60 promoter. The strain carrying the Hsp60 vector was used as a negative control. Bacterial housekeeping gene GroEL2 was used as the loading control. C. Quantification of relative band intensities in D by densitometry. D. Real time RT-PCR to compare transcripts of tdTomato expressed from the L5 promoter with transcripts levels expressed from the Hsp60 promoter. E. Real time q-PCR to compare copy numbers of tdTomato expressing plasmid with the L5 promoter and that carrying the Hsp60 promoter. Data represent one of three replicate experiments. Student’s t-test was performed to examine difference between the L5-tdTomato and Hsp60-tdTomato strains in fluorescence of extracted protein, transcript numbers and gene copies. * represents *P*<0.05; and ** represents *P*<0.01.

We investigated the reasons that the *M*. *bovis* BCG L5-tdTomato strain displays higher fluorescence intensity than the Hsp60-tdTomato strain. At first, protein levels present in the L5-tdTomato and Hsp60-tdTomato strains were compared. Cell lysates of BCG-L5-tdTomato and BCG-Hsp60-tdTomato were evaluated using 100 μg of total protein with a multimode plate reader (Fig 8A). The L5-tdTomato strain had two- to three-fold higher fluorescence than the Hsp60-tdTomato strain. We also compared these strains by Western analyses using antibody against RFP for tdTomato and anti-GroEL2 as a control for protein levels. As shown in Fig 8B and 8C, the tdTomato protein expressed from the L5 promoter is approximately two-fold higher than the level expressed from the Hsp60 promoter. Quantitative real-time RT-PCR was used to compare the transcript levels for the tdTomato gene expressed from the L5 and Hsp60 promoters. As shown in Fig 8D, the tdTomato transcript number when expressed from the L5 promoter is also less than two-fold higher than that expressed from the Hsp60 promoter. Subsequently, gene copy numbers in the two strains were also compared with DNAs as templates using real-time qPCR. Interestingly, the strain having L5 promoter had four-fold higher plasmid copy number than did the strain having Hsp60 promoter (Fig 8E). We conclude that the higher fluorescence intensity of L5-tdTomato strain than the Hsp60-tdTomato strain is a result of more plasmid copy numbers in the L5-tdTomato strain than in the Hsp60-tdTomato strain. Interestingly, despite the four-fold higher copy number, only two-fold higher transcript and proteins levels are observed, suggesting that higher protein levels are blocked.

## Discussion

The slow growth of *M*. *tuberculosis* has hindered research toward discovery and translational development of novel anti-TB therapeutics and vaccines. Noninvasive *in vivo* imaging technologies are emerging for quantification of bacterial loads in animals and has the potential to greatly accelerate TB research. We examined *M*. *bovis* BCG strains expressing various FPs to select an optimal FP and promoter to allow detection of mycobacteria *in vitro* and *in vivo* with fluorescence imaging. Among the selected FPs, we found that tdTomato expressing strains display the greatest fluorescence as compared to other FP expressing strains. When expressed from the L5 promoter, there is a higher level of expression of tdTomato than from the Hsp60 promoter. Interestingly, when we compared the plasmid DNA copy number between the two strains, the L5 promoter strain had a higher copy number for tdTomato, which is four-fold higher than the copy number in the Hsp60 promoter strain. We speculate that tdTomato may be somewhat toxic if overexpressed in the bacteria. Although the L5 promoter strain carries more gene copies than the Hsp60 promoter strain, the L5 promoter strain only expresses slightly higher tdTomato transcript numbers, minimizing the toxicity of tdTomato to the bacterium. To our knowledge, this is the first report of mechanisms responsible for fluorescence intensity differences between FP-labeled bacteria that are due to FP copy number levels at the protein, RNA and DNA level. The results provide insight into strategies for design and construction of FP expressing vectors. To select FP for *in vivo* imaging, they must be examined not only for brightness and excitation and emission wavelengths, but also for the maximal FP expression levels possible in the particular bacterium carrying the construct.

As previously shown by Carroll *et al* [10] and Zelmer *et al* [14], the fluorescent signal in our FP labeled bacteria correlates very well with bacterial numbers by CFU, suggesting that the L5-tdTomato expressing strain can be used to quantify bacterial numbers *in vitro* and *in vivo* immediately, allowing the potential to replace conventional CFU numeration for quantification of bacterial numbers during infection. This tdTomato expressing strain also offers the opportunity to more rapidly evaluate new TB therapeutic regimen *in vitro* and *in vivo*. As the lowest number of bacteria can be detected with this method is 6.5X10^4^ CFU *in vivo*, other methods with better sensitivities should be applied when quantification of bacteria lower than this limit is required in therapy efficacy evaluation.

Among all the selected fluorescent proteins, tdTomato displays the greatest fluorescence, 5.9-fold that of mCherry [33, 39]. The structure of tdTomato is a tandem dimer of mCherry with a few single nucleotide changes [39]. Although tdTomato’s maximum excitation and emission wavelength are lower than 600 nm, which is outside the “NIR window”, the sensitivity for detection of tdTomato expressing mycobacteria is greater than that of mCherry expressing mycobacteria *in vivo*. The greater brightness of tdTomato is the most likely explanation for this difference. The brightness of tdTomato ensures that hemoglobin and water within mammalian tissue only absorb a portion of the excitation light and emission signal.

Both luciferase and fluorescent protein labeled mycobacterial strains have been developed for TB studies [10, 11, 13–19, 40]. Three of these reporter systems have been applied to *in vivo* detection of *Mtb* infection in mice [11, 14, 18, 19]. The sensitivity of our tdTomato expressing strain is similar to that using a luciferase expressing strain [11]. However, we have developed another *in vivo* imaging strategy designated reporter enzyme fluorescence (REF), in which mycobacteria are detected using a fluorogenic substrate for the mycobacterial beta-lactamase. With a REF substrate, CNIR5, we can detect 10^4^ CFU of mycobacteria in lungs of live mice [12]. Both our tdTomato expressing *M*. *bovis* BCG strain and the luciferase expressing *Mtb* strain are somewhat less sensitive than REF imaging of *Mtb* infection *in vivo*. Unlike REF- and luciferase-based imaging, imaging with FP expressing strains do not require administration of a substrate, or time for the substrate to reach peak levels in tissues. These characteristics offer the advantage that our FP reporter strains can be repeatedly imaged over very short periods of time, without having to wait for clearance of previous imaging probes. If brighter fluorescent proteins with longer excitation and emission wavelengths can be developed, the sensitivity of imaging with FP expressing strains could reach that of REF technologies, but still would require recombinant strains, which have their inherent limitations. These limitations could include the inability to image clinical strains without recombinant modification, potential impacts on virulence of pathogenic strains and potential toxicity due to overexpression of reporters.

Another imaging technique that does not require substrate uses the bacterial *luxCDABE* operon, which includes both luciferase and luciferase substrate genes. Mycobacterial strains expressing *luxCDABE* have been used to evaluate anti-TB therapy efficacies [15, 16, 18, 40]. It has been reported that high level expression of *luxD* is toxic to mycobacteria, because *luxD* encodes an acyl transferase involved in bacterial cell wall fatty acid modification [19]. This explains why all mycobacterial whole lux-operon based strains developed to date have incorporated a single copy of the operon in the mycobacterial chromosome [15, 18, 19]. We did not find that expression of tdTomato affected bacterial growth under laboratory culture conditions.

We used strains carrying plasmids expressing multi-copy FP genes. It has been reported that plasmids carrying FP in mycobacterial strains are stable over days to weeks [10]. Our results demonstrate that the tdTomato expressing strain can stably maintain the plasmid for as long as 18 days without antibiotic selective pressure, as has been shown by others previously [10, 14]. This feature makes it suitable for long term *in vitro* and *in vivo* experiments, even without administration of the antibiotic. Our ongoing studies are designed to ensure that mycobacteria express these FPs in a more stable manner using single copy tdTomato and mCherry expressing constructs that are site-specifically integrated into the mycobacterial chromosome, using a mycobacterial plasmid containing the phage attachment site *attP* and the L5 integrase (*int*) gene from the mycobacteriophage L5 genome. These constructs integrate tdTomato or mCherry into the mycobacterial chromosome via site-specific recombination of the plasmid-borne *attP* site with the chromosomal *attB* site, as has been shown by others for bioluminescent reporters [11, 19]. Although these strains carry single copy of fluorescent protein genes, it may not necessary lead to lower fluorescence than strains carrying more copies of fluorescent protein genes. A previous study compared luminescence among *Mtb* strains carrying higher copy, low copy, and single copy chromosomal luminescent genes, and revealed that the highest luminescence was expressed in strains carrying single copy luminescent genes [19]. Similarly, another study compared *Mtb* strains expressing high and low copy of fluorescent protein genes, and found that fluorescence from the higher copy strains were not higher than that from lower copy strains [10]. With the strains carrying FPs in chromosome, long-term animal or cell infection experiments could be conducted in the absence of plasmid selection.

In summary, in agreement with a previous study by others [14] we have demonstrated that *in vivo* fluorescence imaging with FP-expressing mycobacterial strains can be used to quantify bacterial load in animals, which, once more widely applied, could significantly accelerate TB research, particular for analysis of novel therapies and during vaccine development.

## Supporting Information

S1 Fig Comparison of fluorescence of *M*. *smegmatis* expressing Hsp60-tdTomato or Hsp60-mCherry in tubes under sliced ham. This setup was used to optimize the imaging conditions for detecting these two FP expressing strains. Each strain was loaded into eppendorf tubes and imaged with an IVIS Lumina. Eppendorf tubes were covered with various layers of sliced ham and imaged to assess the transmission properties of the fluorescent reporters when scattered by mammalian tissue that contains hemoglobin (ham). Relative fluorescence ratios are shown next to the sites of Eppendorf tubes. They were calculated by setting the sample with the lowest fluorescence as a reference. The experiment was performed once.
(TIFF)

S2 Fig Comparison of fluorescence levels between anti-TB therapy treated and untreated groups. A. Images of INH+RIF treated and untreated BCG-L5-tdTomato strain in culture medium at 96-h post-treatment. 10^6^, 10^5^, and 10^4^ colony forming unites (CFU) were loaded into 96-well plates. Images were acquired by reflective illumination and analyzed with spectral unmixing. B. Quantitative analysis of A. Data represents one of at least three independent replicate experiments. Two-way ANOVA was performed to examine overall difference between the two groups (treated vs. untreated) through multiple CFU groups. Comparison of treated with untreated group was matched at each CFU, *P*<0.001. Bonferroni post-hoc tests were performed by comparison of treated group with untreated group at each CFU group. * represents *P*<0.05; and *** represents *P*<0.001.
(TIFF)
