# Supplementary figures and images for: Application of Fluorescent Protein Expressing Strains to Evaluation of Anti-Tuberculosis Therapeutic Efficacy In Vitro and In Vivo
Source: PLoS One. 2016 Mar 2;11(3):e0149972. doi: 10.1371/journal.pone.0149972 (PMC4774912; doi:10.1371/journal.pone.0149972)

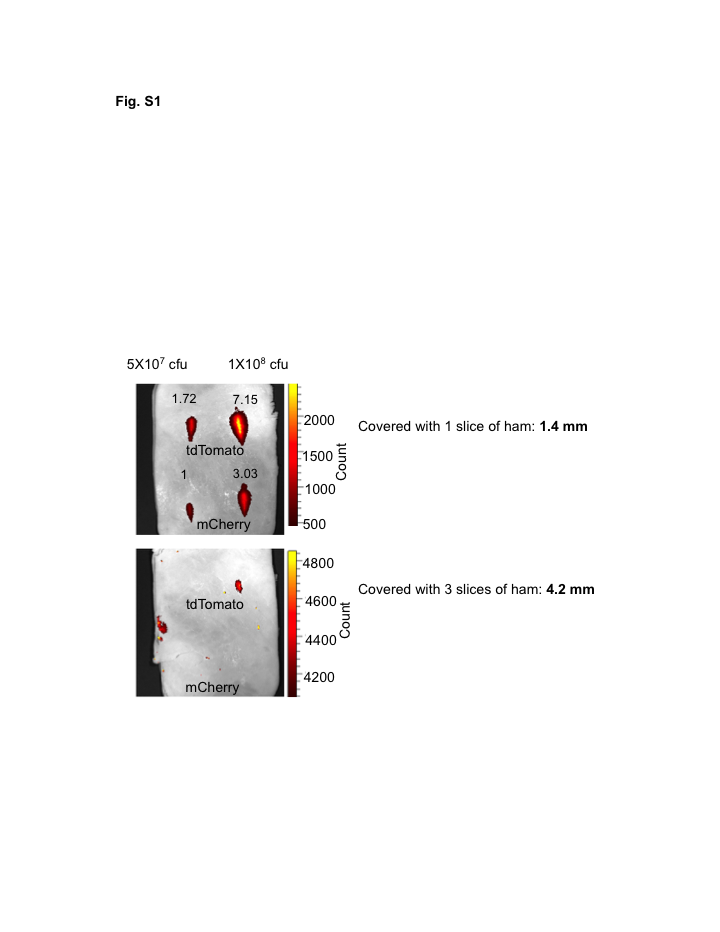

Supplement: S1 Fig — This setup was used to optimize the imaging conditions for detecting these two FP expressing strains. Each strain was loaded into eppendorf tubes and imaged with an IVIS Lumina. Eppendorf tubes were covered with various layers of sliced ham and imaged to assess the transmission properties of the fluorescent reporters when scattered by mammalian tissue that contains hemoglobin (ham). Relative fluorescence ratios are shown next to the sites of Eppendorf tubes. They were calculated by setting the sample with the lowest fluorescence as a reference. The experiment was performed once. (TIFF) [file pone.0149972.s001.tiff]

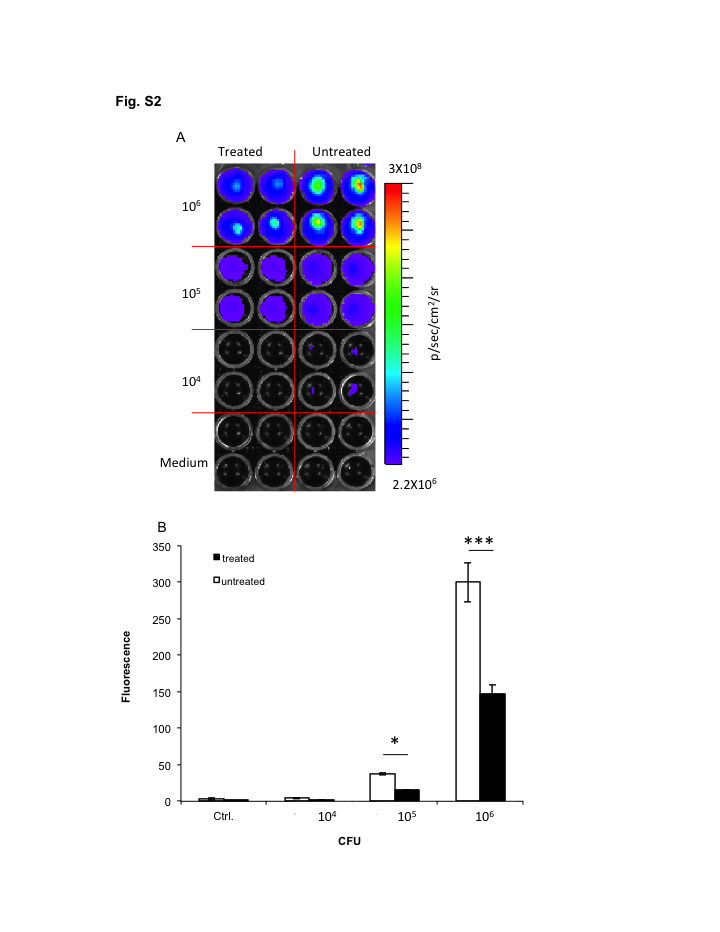

Supplement: S2 Fig — A. Images of INH+RIF treated and untreated BCG-L5-tdTomato strain in culture medium at 96-h post-treatment. 106, 105, and 104 colony forming unites (CFU) were loaded into 96-well plates. Images were acquired by reflective illumination and analyzed with spectral unmixing. B. Quantitative analysis of A. Data represents one of at least three independent replicate experiments. Two-way ANOVA was performed to examine overall difference between the two groups (treated vs. untreated) through multiple CFU groups. Comparison of treated with untreated group was matched at each CFU, P<0.001. Bonferroni post-hoc tests were performed by comparison of treated group with untreated group at each CFU group. * represents P<0.05; and *** represents P<0.001. (TIFF) [file pone.0149972.s002.tiff]
